# Highly Pathogenic Avian Influenza Viruses and Generation of Novel Reassortants, United States, 2014–2015

**DOI:** 10.3201/eid2207.160048

**Published:** 2016-07

**Authors:** Dong-Hun Lee, Justin Bahl, Mia Kim Torchetti, Mary Lea Killian, Hon S. Ip, Thomas J. DeLiberto, David E. Swayne

**Affiliations:** US Department of Agriculture Agricultural Research Service, Athens, Georgia, USA (D.-H. Lee, D.E. Swayne);; University of Texas School of Public Health, Houston, Texas, USA (J. Bahl);; US Department of Agriculture Animal and Plant Health Inspection Service, Ames, Iowa, USA (M.K. Torchetti, M.L. Killian);; US Department of the Interior US Geological Survey, Madison, Wisconsin, USA (H.S. Ip);; US Department of Agriculture Animal and Plant Health Inspection Service, Fort Collins, Colorado, USA (T.J. DeLiberto)

**Keywords:** highly pathogenic avian influenza virus, reassortant, reassortment, North America, waterfowl, bird, migration, phylogenetic analysis, clade 2.3.4.4, viruses, United States, HPAI, influenza

## Abstract

Asian highly pathogenic avian influenza A(H5N8) viruses spread into North America in 2014 during autumn bird migration. Complete genome sequencing and phylogenetic analysis of 32 H5 viruses identified novel H5N1, H5N2, and H5N8 viruses that emerged in late 2014 through reassortment with North American low-pathogenicity avian influenza viruses.

Highly pathogenic avian influenza (HPAI) viruses cause systemic infection and high mortality in poultry species and belong to either the H5 or H7 hemagglutinin (HA) subtypes. In particular, the Asian-origin influenza A(H5N1) A/goose/Guangdong/1/1996 (Gs/GD) lineage of HPAI viruses has become widespread across 4 continents, affecting poultry, wild birds, and humans (*1*).

The H5N1 HPAI virus has evolved into 10 genetically distinct virus clades (0–9) and subclades (*2*). During 2005–2006, clade 2.2 viruses spread from Qinghai Lake, China, to countries across Asia, Europe, and Africa (*3*). Since 2008, HPAI viruses bearing the HA gene of the Gs/GD lineage H5 clade 2.3.4 with N2, N5, and N8 neuraminidase (NA) subtypes have been identified in mainland China (*4*,*5*). In early 2014, outbreaks of novel reassortant H5N6 viruses of clade 2.3.4.4 HA were reported in China, Laos, and Vietnam (*6*) and of H5N8 viruses of the same clade in Japan and South Korea (*7*). Subsequently, H5 clade 2.3.4.4 HPAI viruses originating in East Asia were detected in countries of Asia and Europe and, in late 2014, in North America (*8*). Since first being identified in the Pacific Northwest of the United States, HPAI viruses have been detected in 21 states. Approximately 7.5 million turkeys and 42.1 million chickens have died or have been depopulated as a result (https://www.aphis.usda.gov/wps/portal/aphis/ourfocus/animalhealth/sa_animal_disease_information).

In this study, we conducted a comparative phylogenetic analysis of 32 newly sequenced H5 clade 2.3.4.4 HPAI viruses identified in the United States, including 2 H5N1, 12 H5N2, and 18 H5N8 viruses, to estimate the evolutionary history and to elucidate diversification patterns since emergence in North America. The methods used are detailed in Technical Appendix 1).

Phylogenetic analyses confirmed the wide geographic dispersion of Gs/GD-lineage H5 clade 2.3.4.4 HPAI viruses since late 2014 and movement of this virus from East Asia to North America, West Asia, and Europe (Technical Appendix 1 Figure 1). High bootstrap values (>70%) and long branches in the HA phylogeny supported the delineation of these viruses into 4 groups (Technical Appendix 1 Figure 2). Group intercontinental A (icA) comprises H5N8 viruses identified from China in early 2014 and South Korea, Japan, Taiwan, Canada, the United States, and European countries. The estimated time to most recent common ancestor (tMRCA) was June 2013 (95% Bayesian credible interval [BCI] April–October 2013). Group icA includes reassortant H5N2 and H5N3 viruses from Taiwan and H5N1 and H5N2 viruses from North America. Group B comprises H5N8 viruses identified from China in 2013 and Korea in 2014 (tMRCA April 2013, 95% BCI October 2012–August 2013). Group C comprises H5N6 viruses identified from China and Laos during 2013–2014 and H5N1 viruses identified from China and Vietnam in 2014 (tMRCA November 2012, 95% BCI March 2012–May 2013). Group D comprises H5N6 viruses identified from China and Vietnam during 2013–2014, including isolates from infected humans (A/Sichuan/26221/2014[H5N6] and A/Guangzhou/39715/2014 [H5N6]) (tMRCA September 2012, 95% BCI February 2012–February 2013). These H5 reassortant viruses were descendants of clade 2.3.4 H5N1 viruses identified in 2005 (Technical Appendix 1 Figure 1).

Previous studies reported novel reassortant H5N1 and H5N2 viruses of group icA (*9*,*10*); the H5N1 and H5N2 viruses we sequenced in this study in had identical genome constellations (Figure; Technical Appendix 1 Figures 3–5). Reassortment events after the initial introduction of a group icA H5N8 virus to low-pathogenicity avian influenza (LPAI) viruses led to the divergence of H5 viruses into distinct subtypes, including H5N1, H5N2, and reassortant H5N8. Sixteen H5N8 viruses sequenced in this study had identical genome constellations with previously reported H5N8 viruses from East Asia. In addition, 2 H5N8 reassortant isolates identified from Oregon in January 2015 (A/American wigeon/Oregon/AH0012525/2015 and A/Canada goose/Oregon/AH0012452/2015) had polymerase basic 1 and polymerase acidic genes derived from North American lineage LPAI viruses that did not cluster with the H5N1 and H5N2 reassortant viruses (Technical Appendix 1 Figure 5). Ongoing analysis of existing wild bird surveillance data might aid in filling in the relatively long horizontal branches of the NA and internal genes of H5 reassortant viruses derived from North American LPAI viruses. The occurrence of multiple reassortment events means that group icA H5N8 virus was infecting the same wild birds that were infected with North American LPAI viruses but also that the tissue tropism of Asian H5N8 HPAI and North American LPAI viruses were overlapping, most likely in the cells lining the respiratory and intestinal tract (*11*).

The estimated tMRCA of H5 viruses identified in the United States was October 2014 (95% BCI July–November 2014). The estimated tMRCA of reassortant viruses identified in the United States was December 2014 for H5N1 (95% BCI December 2014–December 2014), November 2014 for H5N2 (95% BCI October 2014–November 2014), and December 2014 for H5N8 (95% BCI November 2014–January 2015) (Table). The tMRCA of H5N8 viruses corresponded to the autumn bird migration season, supporting the hypothesis that Eurasian H5N8 clade 2.3.4.4 virus spread via migratory birds (*8*,*12*,*13*). Subsequently, H5N2 reassortant viruses emerged in November 2014, and H5N1 and H5N8 reassortant viruses emerged in December 2014 (Table; Technical Appendix 1 Figures 3–5).

**Table:** tMRCA for H5 highly pathogenic avian influenza viruses, by gene, United States, 2014–2015

Gene	tMRCA (95% BCI, posterior probability)			
	H5N8	H5N8 reassortant	H5N1 reassortant	H5N2 reassortant
HA	Oct 2014 (Jul 2014–Nov 2014, 0.81)	Dec 2014 (Nov 2014–Jan 2015, 0.67)	Dec 2014 (Dec 2014–Dec 2014, 1.00)	Nov 2014 (Oct 2014–Nov 2014, 0.99)
NA	Jul 2014 (Feb 2014–Nov 2014, 0.76)	Dec 2014 (Nov 2014–Jan 2015, 1.00)	Mar 2014 (Jul 2013–Oct 2014, 1.00)	Sep 2014 (Jun 2014– Nov 2014, 1.00)
PB2	Oct 2014 (Aug 2014–Nov 2014, 1.00)	Dec 2014 (Oct 2014– Jan 2015, 0.48)	Nov 2014 (Oct 2014–Dec 2014, 0.95)	Nov 2014 (Oct 2014– Nov 2014, 0.99)
PB1	Oct 2014 (Jul 2014–Nov 2014, 1.00)	Dec 2014 (Nov 2014–Jan 2015, 1.00)	Dec 2014 (Nov 2014–Dec 2014, 1.00)	Oct 2014 (Aug 2014– Nov 2014, 1.00)
PA	Sep 2014 (Jul 2014–Nov 2014, 0.38)	Nov 2014 (Sep 2014–Jan 2015, 1.00)	Nov 2014 (Oct 2014–Dec 2014, 0.98)	Oct 2014 (Sep 2014–Nov 2014, 1.00)
NP	Jul 2014 (Mar 2014–Nov 2014, 0.53)	Nov 2014 (Jul 2014–Jan 2015, 0.67)	Nov 2014 (Oct 2014–Dec 2014, 1.00)	Nov 2014 (Jun 2014–Nov 2014, 1.00)
M	Jul 2014 (Jan 2014–Dec 2014, 0.95)	Nov 2014 (Jun 2014–Jan 2015, 0.38)	Dec 2014 (Nov 2014–Dec 2014, 1.00)	Aug 2014 (Mar 2014–Nov 2014, 0.34)
NS	May 2014 (Nov 2013–Nov 2014, 0.08)	Dec 2014 (Nov 2014–Jan 2015, 1.00)	Nov 2014 (Sep 2014–Dec 2014, 1.00)	May 2014 (Oct 2013–Oct 2014, 0.86)

*BCI, Bayesian credible interval; HA, hemagglutinin; M, membrane; NA, neuraminidase; NP, nucleoprotein; NS, nonstructural; PA, polymerase acidic; PB1, polymerase basic 1; PB2, polymerase basic 2; tMRCA, time to most recent common ancestor.

Wild bird migration and illegal trade of infected poultry, eggs, and poultry products have caused the spread of HPAI viruses (*14*). The South Korea H5N8 outbreak in January 2014 was the first H5N8 virus reported outside of China. Wild migratory birds were suspected to play a key role in the introduction of group icA and B viruses from eastern China and in the subsequent spread during the 2014 South Korea outbreak (*15*). Previous studies hypothesized that wild bird migration might play a role in dispersal of these viruses; the H5N8 virus was identified in a long-distance migrant bird (Eurasian wigeon, *Anas penelope*) in eastern Siberia in September 2014 and subsequently in multiple wild bird species in Japan, Europe, and the west coast of North America in November and December 2014 (*8*,*12*). In contrast, group C H5N6 HPAI viruses in Laos were most likely transmitted by live poultry imports from China (*6*).

The continued reassortment of H5 clade 2.3.4.4 HPAI viruses with co-circulating HPAI and LPAI viruses created a diverse genetic pool of H5 clade 2.3.4.4 that has spread to various countries. This contrasts with the expansion of H5N1 clade 2.2 from Asia to Western Europe during 2005–2006, when such frequent reassortment was not recorded. In eastern China, H5N2 HPAI viruses isolated in 2011 were generated from reassortment events in which the neuraminidase and nonstructural gene segments of H5N1 HPAI viruses were replaced with those derived from locally circulating LPAI viruses (*4*). The H5N8 viruses of group B had polymerase basic 2, neuraminidase, and nonstructural genes derived from local LPAI viruses (*5*). The H5N6 viruses of group C identified in Laos were generated through reassortment between H5N1 viruses from clade 2.3.2.1b, clade 2.3.4, and H6N6 LPAI viruses that circulate broadly in duck populations in China (*6*).

H5 clade 2.3.4.4 viruses have spread globally through wild bird migration and the poultry trade (*6*,*8*,*12*,*13*). In addition, these viruses generated a variety of reassortant viruses that shuffled genes with prevailing local viruses. The continued circulation of HPAI viruses in wild and domestic avian populations contributes to the persistence and diversity of circulating avian influenza viruses. Enhanced active surveillance provides the opportunity to monitor the spread and reassortment of clade 2.3.4.4 and to fortify the biosecurity of farms in affected regions.

**Figure:**
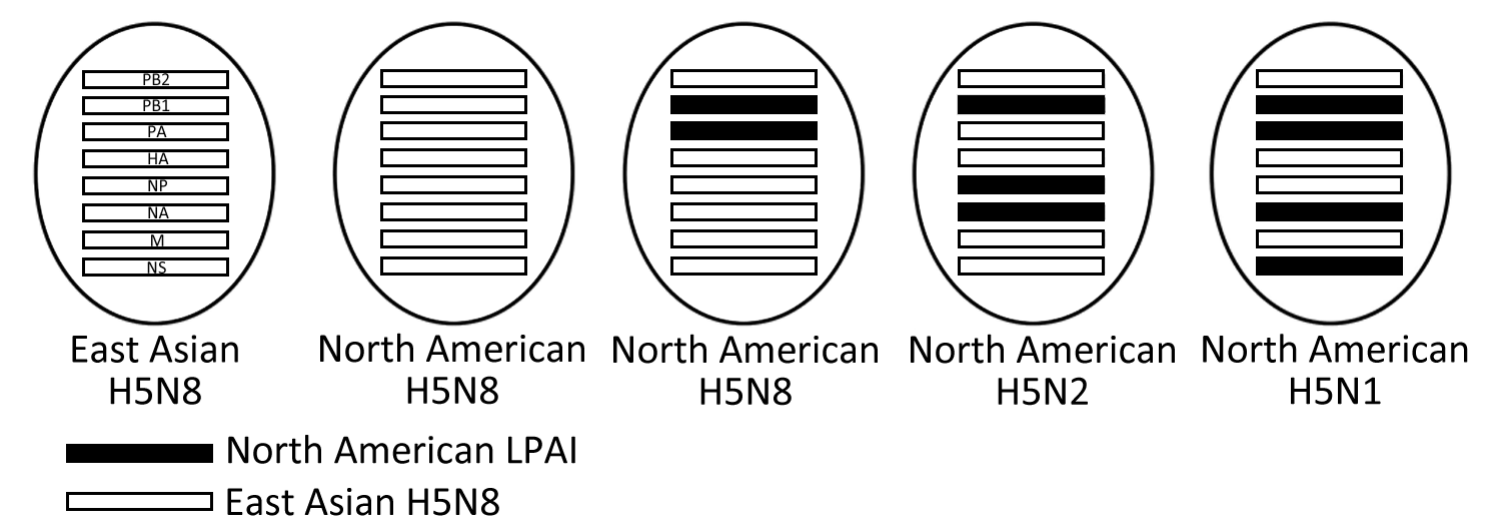
Schematic diagram of the H5 clade 2.3.4.4 highly pathogenic avian influenza virus genotypes identified in this study, United States, 2014–2015. Reassortant H5N8 comprises Eurasian PB2, PA, HA, NP, M, and NS gene segments, and North American PB1 and PA gene segments; reassortant H5N2 comprises Eurasian PB2, PA, HA, M, and NS gene segments, and North American NA, PB1, and NP gene segments; reassortant H5N1 comprises Eurasian HA, NP, M, and PB2 gene segments and North American NA, NS, PA, and PB1 gene segments. HA, hemagglutinin; LPAI, low-pathogenicity avian influenza; M, matrix; NA, neuraminidase; NP, nucleoprotein; NS, nonstructural; PA, polymerase acidic; PB1, polymerase basic 1; PB2, polymerase basic 2.

Technical Appendix 1 Methods for genome sequencing and phylogenetic analysis.

Technical Appendix 2 Acknowledgment of contributions of colleagues through the Global Initiative on Sharing All Influenza Data.

## References

[1] Swayne DE, Suarez DL, Sims LD. Influenza. In: Swayne DE, Glisson JR, McDougald LR, Nair V, Nolan LK, Suarez DL, editors. Diseases of poultry, 13th edition. Ames (Iowa): Wiley-Blackwell; 2013. p. 181–218.

[2] WHO/OIE/FAO H5N1 Evolution Working Group. Toward a unified nomenclature system for highly pathogenic avian influenza virus (H5N1). Emerg Infect Dis. 2008;14:e1 . 10.3201/eid1407.071681 PMC2600337 18598616

[3] Salzberg SL, Kingsford C, Cattoli G, Spiro DJ, Janies DA, Aly MM, Genome analysis linking recent European and African influenza (H5N1) viruses. Emerg Infect Dis. 2007;13:713–8. 10.3201/eid1305.070013 17553249 PMC2432181

[4] Zhao G, Gu X, Lu X, Pan J, Duan Z, Zhao K, Novel reassortant highly pathogenic H5N2 avian influenza viruses in poultry in China. PLoS One. 2012;7:e46183. 10.1371/journal.pone.0046183 23049973 PMC3458027

[5] Wu H, Peng X, Xu L, Jin C, Cheng L, Lu X, Novel reassortant influenza A(H5N8) viruses in domestic ducks, eastern China. Emerg Infect Dis. 2014;20:1315–8. 10.3201/eid2008.140339 25075453 PMC4111196

[6] Wong FY, Phommachanh P, Kalpravidh W, Chanthavisouk C, Gilbert J, Bingham J, Reassortant highly pathogenic influenza A(H5N6) virus in Laos. Emerg Infect Dis. 2015;21:511–6. 10.3201/eid2103.141488 25695754 PMC4344285

[7] Lee YJ, Kang HM, Lee EK, Song BM, Jeong J, Kwon YK, Novel reassortant influenza A(H5N8) viruses, South Korea, 2014. Emerg Infect Dis. 2014;20:1087–9. 10.3201/eid2006.140233 24856098 PMC4036756

[8] Lee DH, Torchetti MK, Winker K, Ip HS, Song CS, Swayne DE. Intercontinental spread of Asian-origin H5N8 to North America through Beringia by migratory birds. J Virol. 2015;89:6521–4. 10.1128/JVI.00728-15 25855748 PMC4474297

[9] Torchetti MK, Killian ML, Dusek RJ, Pedersen JC, Hines N, Bodenstein B, Novel H5 clade 2.3.4.4 reassortant (H5N1) virus from a green-winged teal in Washington, USA. Genome Announc. 2015;3:e00195–15. 10.1128/genomeA.00195-15 25838478 PMC4384482

[10] Ip HS, Torchetti MK, Crespo R, Kohrs P, DeBruyn P, Mansfield KG, Novel Eurasian highly pathogenic avian influenza A H5 viruses in wild birds, Washington, USA, 2014. Emerg Infect Dis. 2015;21:886–90. 10.3201/eid2105.142020 25898265 PMC4412248

[11] Webster RG, Yakhno M, Hinshaw VS, Bean WJ, Murti KG. Intestinal influenza: replication and characterization of influenza viruses in ducks. Virology. 1978;84:268–78. 10.1016/0042-6822(78)90247-7 23604 PMC7131577

[12] Verhagen JH, Herfst S, Fouchier RA. Infectious disease. How a virus travels the world. Science. 2015;347:616–7. 10.1126/science.aaa6724 25657235

[13] Saito T, Tanikawa T, Uchida Y, Takemae N, Kanehira K, Tsunekuni R. Intracontinental and intercontinental dissemination of Asian H5 highly pathogenic avian influenza virus (clade 2.3.4.4) in the winter of 2014–2015. Rev Med Virol. 2015;25:388–405. 10.1002/rmv.1857 26458727

[14] Suarez DL. Avian influenza: our current understanding. Anim Health Res Rev. 2010;11:19–33. 10.1017/S1466252310000095 20591211

[15] Jeong J, Kang HM, Lee EK, Song BM, Kwon YK, Kim HR, Highly pathogenic avian influenza virus (H5N8) in domestic poultry and its relationship with migratory birds in South Korea during 2014. Vet Microbiol. 2014;173:249–57. 10.1016/j.vetmic.2014.08.002 25192767

