# Supplementary material for: Highly Pathogenic Avian Influenza Viruses and Generation of Novel Reassortants, United States, 2014–2015
Source: Emerg Infect Dis. 2016 Jul;22(7):1283–5. doi: 10.3201/eid2207.160048 (PMC4918163; doi:10.3201/eid2207.160048)
Supplement: Technical Appendix 1 — Methods for genome sequencing and phylogenetic analysis. [file 16-0048-Techapp-s1.pdf]

Article DOI: http://dx.doi.org/10.3201/eid2207.160048

Highly Pathogenic Avian Influenza Viruses and Generation of Novel Reassortants, United States, 2014–2015

Technical Appendix 1

Methods for Genome Sequencing and Phylogenetic Analysis

Nucleotide Sequencing

In this study, we sequenced 32 clade 2.3.4.4 HPAI H5 viruses identified in the United States. Based on epidemiologic investigation data, two H5N1, 12 H5N2 and 18 H5N8 viruses were selected to represent each subtype from West and Mid-west regions of the United States: H5N2 and H5N8 viruses identified from poultry farm in Arkansas, California, Kansas, Minnesota, Missouri, Oregon, and Washington; H5N1 and H5N8 viruses identified from wild birds in Idaho, Nevada, Oregon, Utah, and Washington. Viral RNA was extracted from samples using the MagMAX Viral RNA Isolation Kit (Ambion/ThermoFisher Scientific). Complementary DNA was synthesized by reverse transcription reaction using SuperScript III (Invitrogen/ThermoFisher Scientific). All 8 segments of isolates were amplified by PCR and complete genome sequencing was conducted using the Ion Torrent (Life Technologies) platform. Briefly, the PCR product was purified and DNA libraries were prepared with the IonXpress Plus Fragment Library Kit (Life Technologies) with Ion Xpress barcode adapters. Prepared libraries were quantitated using the Bioanalyzer DNA 1000. Quantitated libraries were diluted and pooled for library amplification using the Ion One Touch 2 and ES systems. Following enrichment, DNA was loaded onto an Ion 314 or Ion 316 chip and sequenced using the Ion PGM 200 v2 Sequencing Kit. De Novo and directed assembly of genome sequences were carried out using the SeqMan NGen v. Four program. Nucleotide sequences for complete genome of 2 H5N1, 12 H5N2 and 18 H5N8 viruses have been deposited in GenBank under accession no. KR234027-KR234034, KR233995-KR234002, KR233987-KR233994, KP739378-KP739385, KP739386-KP739393, KP739410-KP739417, KR150906-KR150913, KR234019-KR234026, KR150898-

KR150905, KR234003-KR234010, KR234035-KR234042, KR233979-KR233986, KR234011-KR234018, KP739402-KP739409, KP739394-KP739401, KU201789-KU201796, KU201797-KU201804, KU201765-KU201772, KU201773-KU201780, KU201685-KU201692, KU201757-KU201764, KU201869-KU201876, KU201837-KU201844, KU201901-KU201908, KU201749-KU201756, KU201613-KU201620, KU201885-KU201892, KU201877-KU201884, KU201821-KU201828, KU201853-KU201860, and KU201861-KU201868

Phylogenetic Analysis

For phylogenetic analysis, nucleotide sequences used in this study were obtained from GenBank (www.ncbi.nlm.nih.gov/genomes/FLU) and GISAID (http://www.gisaid.org, see acknowledgment tables for laboratory contributions in online Technical Appendix 2, http://wwwnc.cdc.gov/EID/article/22/7/16-0048-Techapp2.xlsx). Complete coding sequences of each gene segment were used for comparative genetic analyses. The Bayesian relaxed clock phylogenetic tree and maximum-likelihood (ML) phylogenetic tree of the HA gene were constructed using nucleotide sequences. Nucleotide positions 49 to 1659 of the HA gene were used for phylogenetic analyses. Multiple sequence alignments were prepared using MUSCLE (*1*), and manual optimization of the alignment was done using MEGA 6 (*2*). The ML tree was estimated with the MEGA 6 software package using the Hasegawa-Kishino-Yano model of nucleotide substitution with gamma-distributed rate variation among sites (with four rate categories) with subtree pruning and regrafting branch swapping. Statistical support for the tree topology was determined by bootstrap analysis with 1000 replicates. Bayesian relaxed clock phylogenetic analyses were done using BEAST v1.8.2 (*3*). For the Bayesian relaxed clock phylogenetic tree of HA gene, we applied an uncorrelated lognormal distribution relaxed clock method, the HKY nucleotide substitution model and the Bayesian skyline coalescent prior. We initially reconstructed the phylogenetic tree using all of the clade 2.3.4.4 viruses available in the GenBank and GISAID and selected representative sequences for our dataset. Our final dataset to estimate time of emergence and spread of clade 2.3.4.4 viruses (Figure) and North American H5 viruses (Technical Appendix 1 Figure 3) contained 143 and 84 sequences, respectively. A Markov Chain Monte Carlo (MCMC) method to sample trees and evolutionary parameters was run for 5.0×10^7 generations. At least three independent chains were combined to ensure adequate sampling of the posterior distribution of trees. For inference of the neuraminidase (NA subtypes N1, ntax = 30; N2, ntax = 44; and N8, ntax = 27) and internal gene trees, polymerase

basic 2 (PB2) (ntax = 56), polymerase basic 1 (PB1) (ntax = 57), polymerase acidic (PA) (ntax = 58), nucleoprotein (NP) (ntax = 55), matrix (M) (ntax = 57), and non-structural (NS) (ntax = 58) gene segments identified in North America, we applied an uncorrelated lognormal distribution relaxed clock method, HKY nucleotide substitution model and the Bayesian skyline coalescent prior, and the Bayesian skyline coalescent prior with 10 groups. A MCMC method to sample trees and evolutionary parameters was run for 3.0×10^7 generations. At least three independent chains were combined to ensure adequate sampling of the posterior distribution of trees. BEAST output was analyzed with TRACER v1.4 with 10% burn-in. A maximum clade credibility (MCC) tree was generated for each dataset using TreeAnnotator in BEAST. FigTree 1.4.2 (http://tree.bio.ed.ac.uk) was used for visualization of trees.

References

1. Edgar RC. MUSCLE: multiple sequence alignment with high accuracy and high throughput. Nucleic Acids Res. 2004;32:1792–7. PubMed http://dx.doi.org/10.1093/nar/gkh340

2. Tamura K, Stecher G, Peterson D, Filipski A, Kumar S. MEGA6: Molecular Evolutionary Genetics Analysis version 6.0. Mol Biol Evol. 2013;30:2725–9. PubMed http://dx.doi.org/10.1093/molbev/mst197

3. Drummond AJ, Rambaut A. BEAST: Bayesian evolutionary analysis by sampling trees. BMC Evol Biol. 2007;7:214. PubMed http://dx.doi.org/10.1186/1471-2148-7-214

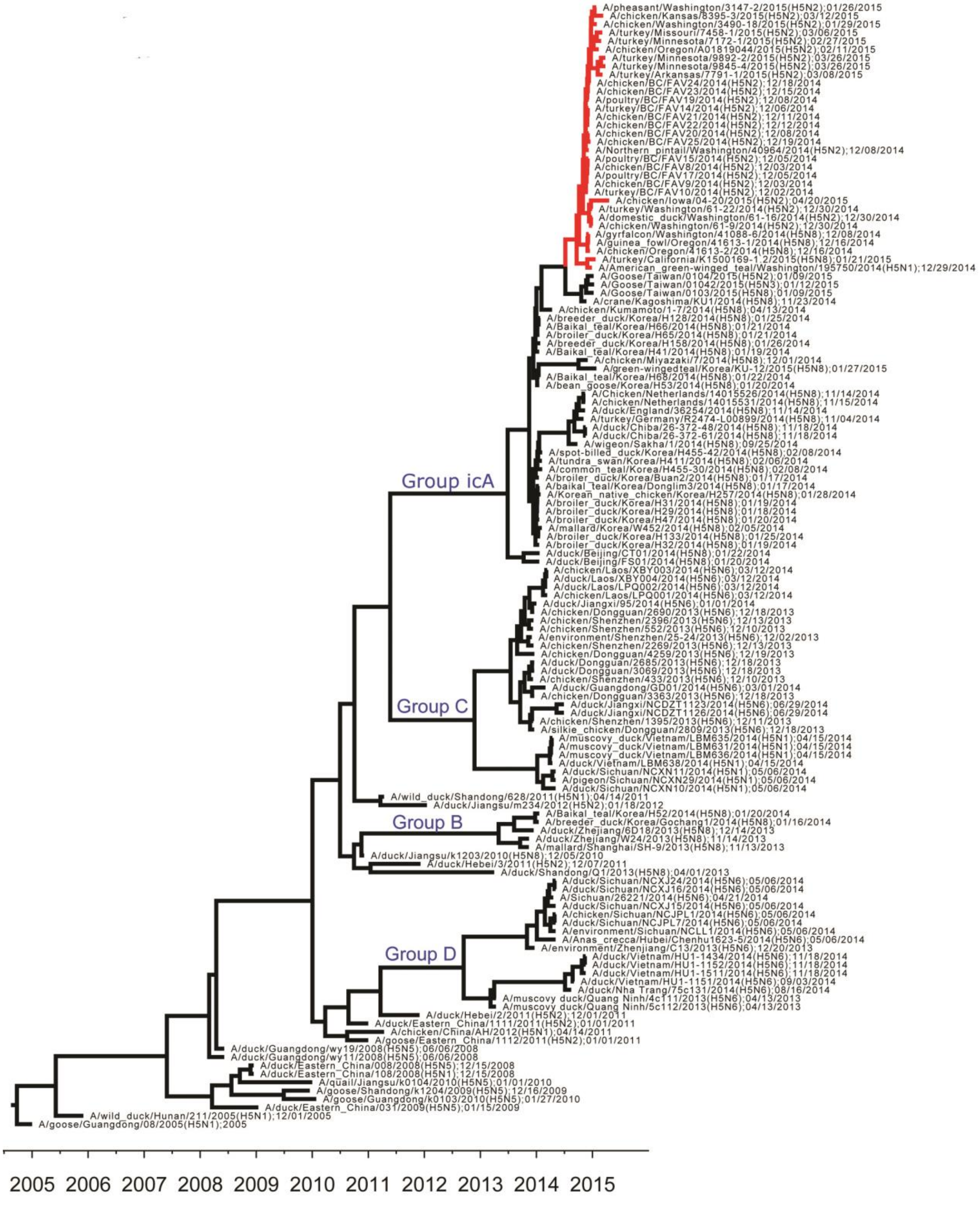

Technical Appendix Figure 1. Relaxed clock molecular phylogenetic tree for the hemagglutinin of H5 clade 2.3.4.4 HPAIV. The phylogenetic relationships and temporal evolutionary history have been estimated by molecular clock analysis. Group designations are indicated at the branch. Red branches represent the H5 clade 2.3.4.4 HPAIV identified in North America.

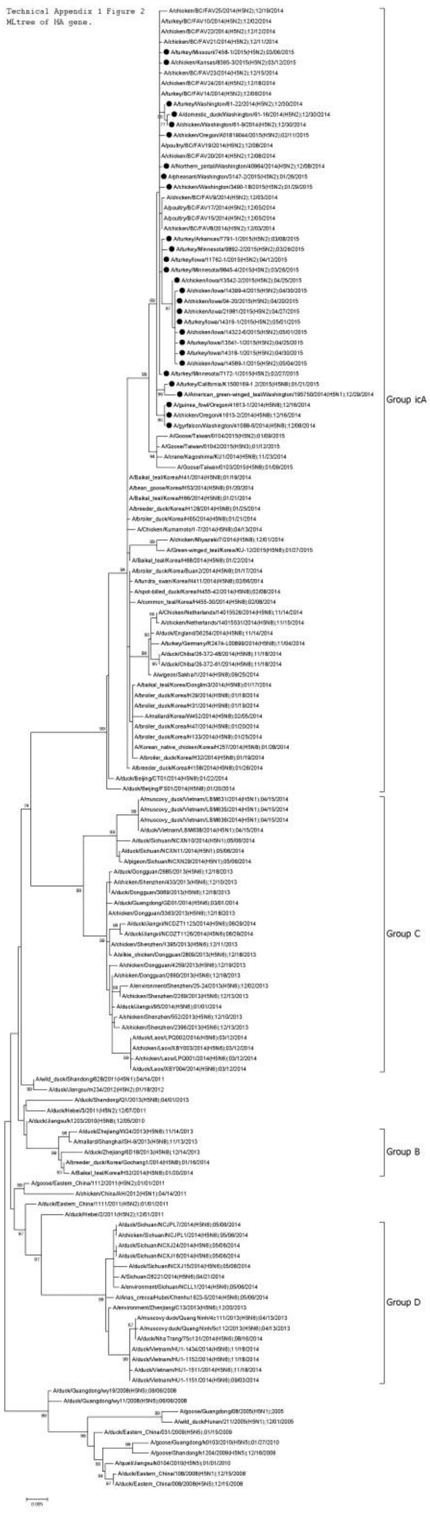

Technical Appendix Figure 2. Maximum-likelihood phylogenetic tree for the hemagglutinin of H5 clade 2.3.4.4 HPAIV. Black circles identify the viruses detected in the United States. At each branch, the number indicates a bootstrap value.

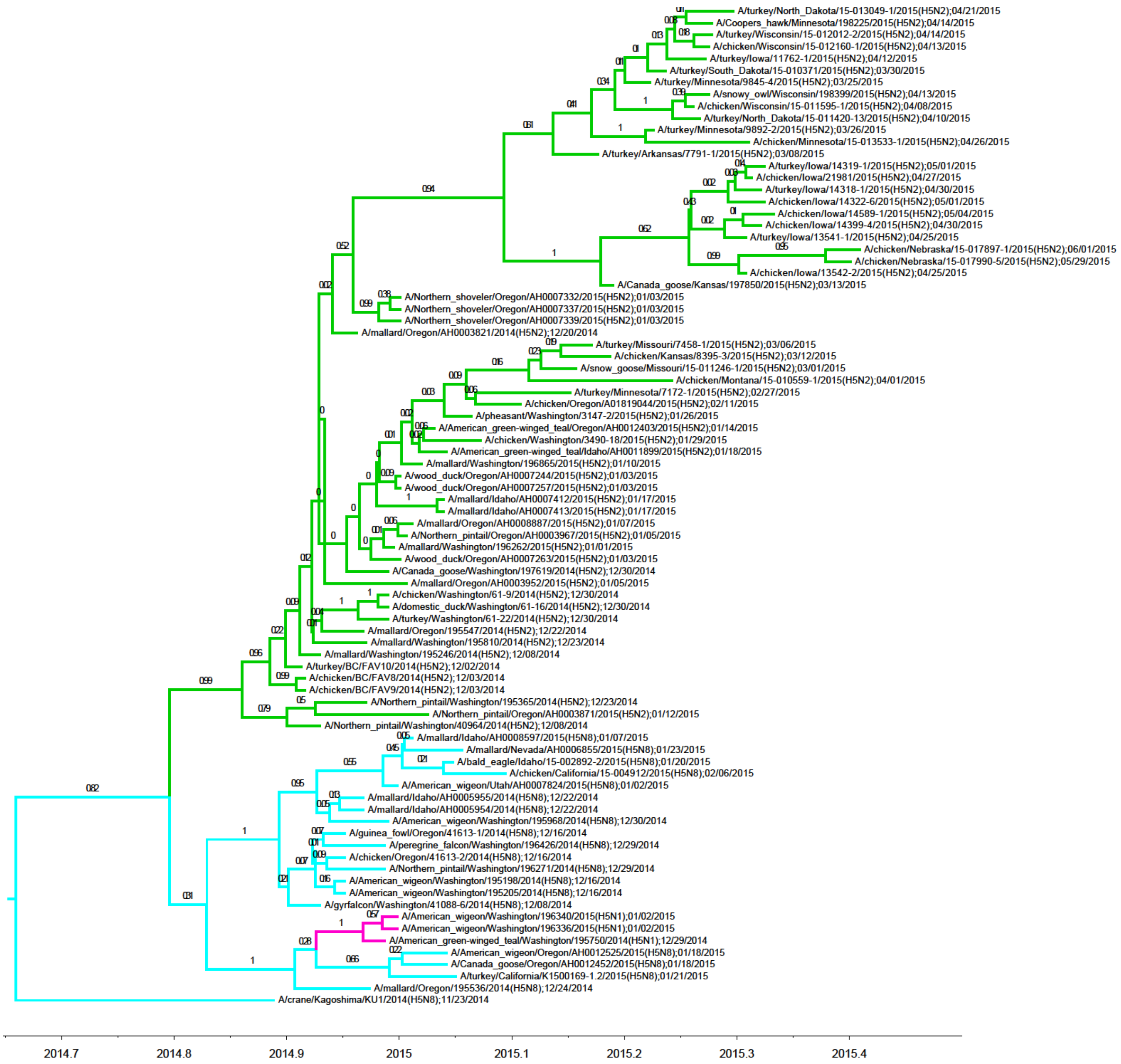

Technical Appendix Figure 3. Relaxed clock molecular phylogenetic tree for the hemagglutinin of H5 clade 2.3.4.4 HPAIV identified in United States. The phylogenetic relationships and temporal evolutionary history have been estimated by molecular clock analysis. The three branch colors are used to distinguish the subtypes: purple for H5N1, green for H5N2, and blue for H5N8. At each branch, the number indicates a posterior probability.

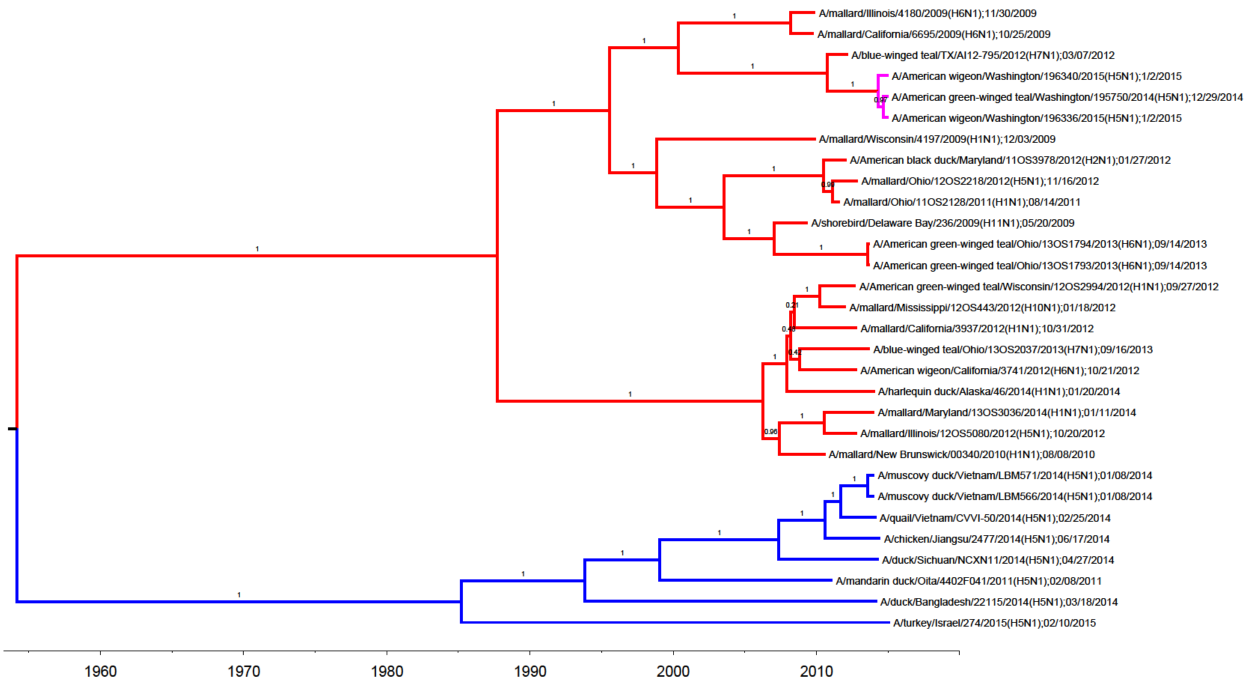

Technical Appendix Figure 4. Relaxed clock molecular phylogenetic tree for the neuraminidase of H5 clade 2.3.4.4 virus HPAIV identified in United States. The phylogenetic relationships and temporal evolutionary history have been estimated by molecular clock analysis. Red and blue branches represent the North American and Eurasian lineage, respectively. The three branch colors are used to distinguish the subtypes: purple for H5N1, green for H5N2, and blue for H5N8. (A) N1, (B) N2, and (C) N8. At each branch, the number indicates a posterior probability.

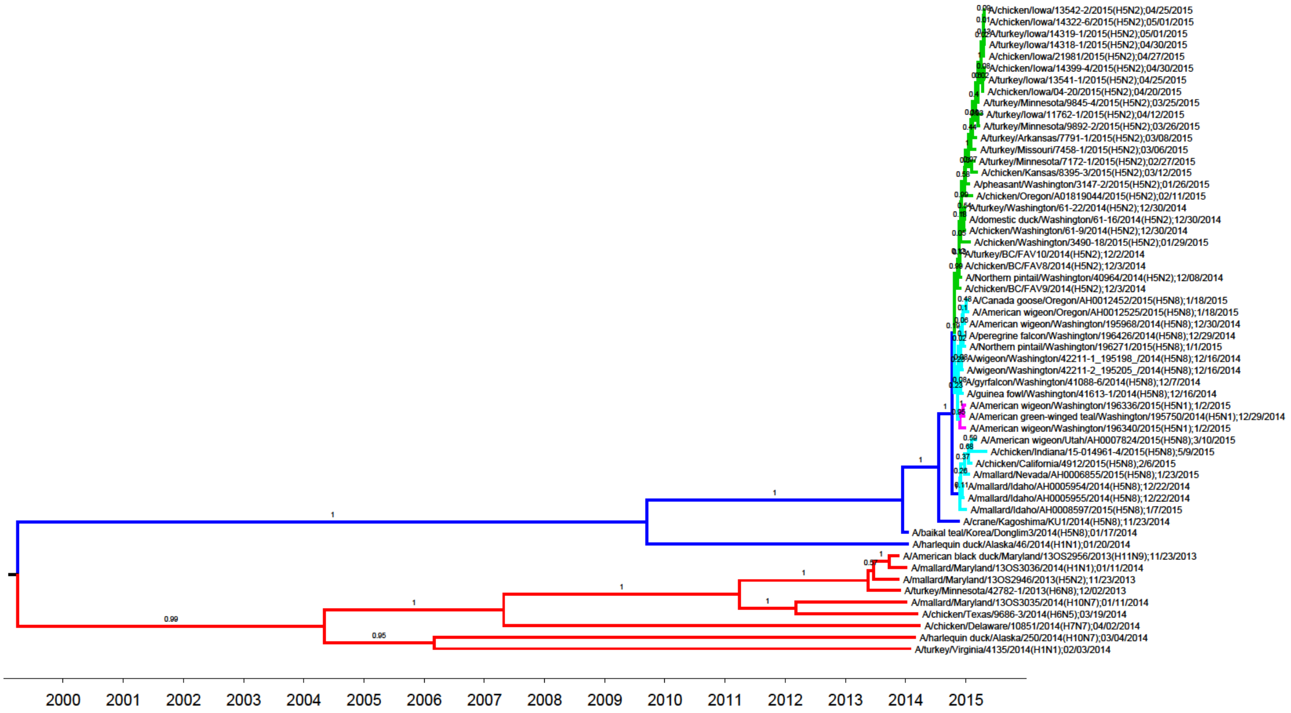

Technical Appendix Figure 5. Relaxed clock molecular phylogenetic tree for the internal genes of H5 clade 2.3.4.4 HPAIV identified in United States. The phylogenetic relationships and temporal evolutionary history have been estimated by molecular clock analysis. Red and blue branches represent the North American and Eurasian lineage, respectively. The three branch colors are used to distinguish the subtypes: purple for H5N1, green for H5N2, and blue for H5N8. (A) PB2, (B) PB1, (C) PA, (D) NP, (E) M, and (F) NS. At each branch, the number indicates a posterior probability.
